# Cervical carcinoma in the setting of uterovaginal prolapse: comparing standard versus tailored management

**DOI:** 10.3332/ecancer.2020.1043

**Published:** 2020-05-13

**Authors:** Ryan M Kahn, Sushmita Gordhandas, Kiersten Craig, Tanaka J Dune, Kevin Holcomb, Eloise Chapman-Davis, Melissa K Frey

**Affiliations:** 1 Department of Obstetrics and Gynecology, Weill Cornell Medicine, New York, NY 10065, USA; 2 Department of Urology, Weill Cornell Medicine, New York, NY 10065, USA; 3 Division of Gynecologic Oncology, Department of Obstetrics and Gynecology, Weill Cornell Medicine, New York, NY 10065, USA; a http://orcid.org/0000-0002-5596-6238

**Keywords:** cervical cancer, PD-L1, uterovaginal prolapse, procidentia

## Abstract

**Objectives:**

Cervical cancer in the setting of uterovaginal prolapse is exceedingly rare. Altered anatomy can complicate treatment of underlying cancer. We first present a rare case of cervical cancer with invasion of uterovaginal prolapse as well as a systematic review of similar reported cases in the literature. The objective of this study was to compare the practice patterns and outcomes regarding cervical cancer with invasion of procidentia.

**Methods:**

We conducted a systematic search of online databases (PubMed, Embase, Medline and the Cochrane Library) from 1990 to 2018 of all the cases of cervical cancer associated with stage III–IV uterovaginal prolapse. Patient demographics, pathology, surgical management, chemotherapy, radiation and disease-free survival were compared.

**Results:**

Fifteen reported cases of cervical cancer in the setting of procidentia were identified (squamous cell carcinoma—14, clear cell carcinoma—1). The mean age at diagnosis was 74 years (range 54–89). Thirteen percent (*n* = 2) of cases presented with FIGO stage I disease, 67% (*n* = 10) with stage II, and 20% (*n* = 3) with stage III. All cases had stage III–IV uterovaginal prolapse. 73% (11) were treated surgically including nine patients who underwent vaginal hysterectomy. Among patients who underwent primary surgery, 7% (1) received adjuvant chemotherapy, 33% (5) adjuvant radiotherapy and 21% (3) both adjuvant chemotherapy and radiation. 33% (5) of surgical cases included procedures to address the pelvic organ prolapse (colpoclesis (*n* = 3), uterosacral suspension (*n* = 1) and anterior posterior repair (*n* = 1)). Two patients died from the disease within 12 months, one patient died from other causes within 1 month, and the remainder of patients were free of disease at last reported follow-up (Table 1).

**Conclusions:**

Cervical cancer in the setting of stage III–IV uterovaginal prolapse can be managed successfully with standard treatment strategies (primary surgery with adjuvant therapy as necessary versus chemoradiation). When patients are surgical candidates, techniques to address the underlying prolapse can be used for symptomatic improvement.

## Introduction

Worldwide, cervical cancer accounts for an estimated 528,000 new cancer cases and 266,000 deaths each year [1]. In under-developed regions, cervical cancer is the second most common type of cancer as well as the third most common cause of cancer related deaths [2]. However, in developed countries with increased screening across the population, cervical cancer represents the 11th most common type of cancer and ninth most common cause of cancer related mortality [3]. The American Cancer society estimates about 13,240 new cases of cervical cancer will be diagnosed in the United States in 2018, with approximately 4,170 deaths from the disease [4]. Pelvic organ prolapse is a far more common disease, estimated to occur in roughly 40%–60% of parous women [5]. The association between cervical cancer and uterovaginal prolapse is an exceedingly rare occurrence in developed countries. There has been minimal literature reported with no current guidelines in regards to the treatment. This is a case-report of cervical carcinoma with invasion of stage IV uterine prolapse and lung metastasis with a full systematic review of the current literature on management patterns and outcomes. This is the first reported case of PD-L1 cervical cancer with invasion of uterovaginal prolapse as well as the first reported case undergoing immunotherapy. Additionally, this is the first complete systematic review performed investigating past cases of cervical cancer associated with pelvic organ prolapse.

## Case report

**Figure 1.**
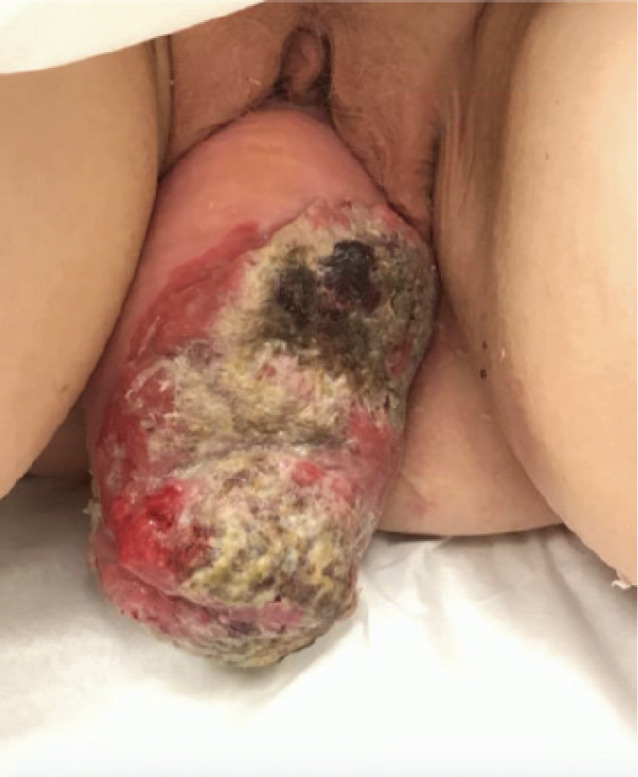
Image of 14 cm × 10 cm necrotic mass associated with stage 4 uterine prolapse on presentation.

This is a 68 year-old post-menopausal, gravida four para three, with a long standing history of uterovaginal prolapse. The patient was unable to describe the duration of her procidentia but reported that the prolapse began to protrude past the vagina for seven months prior to presentation and has been unable to reduce the mass. The patient then began to note a large malodorous, fungating lesion on the prolapse associated with vaginal bleeding. At time of presentation, physical exam revealed a 14 cm × 10 cm necrotic mass (Figure 1).

**Figure 2.**
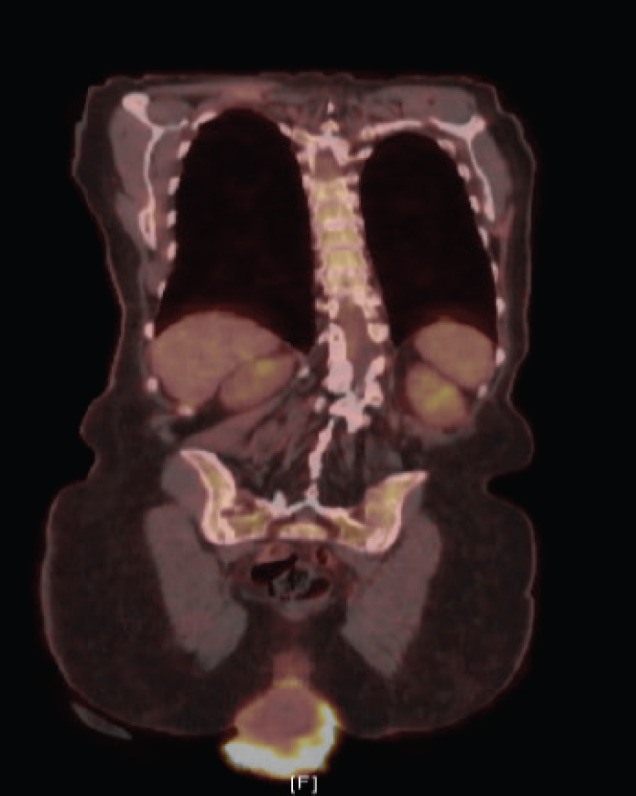
PET/CT demonstrating severe anterior, middle, and posterior compartment prolapse, with protruded mass with peripheral FDG uptake.

A biopsy of the lesion was preformed establishing the diagnosis of invasive squamous cell carcinoma, moderately to poorly differentiated, focally keratinising with a background of high-grade intraepithelial lesion. Computed tomography (CT) of the abdomen and pelvis demonstrated prolapse of part of the bladder, entire uterus and vagina with the gonadal vessels extending inferior to the perineum with two noncalcified lung nodules (8 mm right middle lobe, 5 mm left lower lobe). Follow-up positron emission tomography/computed tomography (PET/CT) showed severe anterior, middle and posterior compartment prolapse with protruded mass with peripheral FDG uptake (Figure 2); hypermetabolic left common iliac, external iliac and obturator lymphadenopathy, few with mild FDG uptake, and mildly FDG avid hilar lymph nodes that are likely reactive.

The patient was diagnosed with stage IIB invasive squamous cell carcinoma of the cervix. Given the finding of PET positive pelvic lymph nodes—along with the size of the tumour—she was not a surgical candidate. The patient underwent four total cycles of weekly Cisplatin 40 mg/m^2^ with 16 fractions of pelvic extended field radiation therapy. The evaluation on each office visit demonstrated areas of resolving necrosis with improvement on each subsequent exam. A follow-up PET/CT 2 months after the completion of therapy demonstrated a decrease in degree of hypermetabolic activity associated with cervical mass hypermetabolic pelvic lymphadenopathy with a new 2.7 cm markedly hypermetabolic right upper lobe nodule suspicious for new metastasis. The patient underwent CT-guided fine-needle aspiration biopsy of the right pulmonary nodule. Pathology demonstrated squamous cell carcinoma, likely metastatic. Immunohistochemistry was p40 positive and strongly p16 positive in support of metastatic disease, HPV was negative. Further testing demonstrated positive PD-L1 and received four cycles of pembrolizumab 200 mg. She also began experiencing worsening, persistent lower back pain. MRI of the lumbar spine demonstrated left lumbar vertebral metastasis. The patient died of disease 12 months following diagnosis.

## Method

This study was registered with PROSPERO, the international prospective register of systematic reviews and followed the guidelines set forth by the preferred reporting items for systematic reviews and meta-analyses (PRISMA) statement.[6] A comprehensive literature search was conducted using the following bibliographic databases: Ovid MEDLINE® (In-Process & Other Non-Indexed Citations and Ovid MEDLINE® 1946 to Present), Ovid EMBASE (1974 to present), and The Cochrane Library (Wiley). There were no language, date, or article type restrictions included in the search.

### Strategy for search of articles and selection criteria

This study was registered with PROSPERO, the international prospective register of systematic reviews and followed the guidelines set forth by the PRISMA statement [6]. A comprehensive literature search was conducted by the institutional medical librarian team on January 11, 2018 using the following bibliographic databases from inception: Ovid MEDLINE® (In-Process & Other Non-Indexed Citations and Ovid MEDLINE® 1946 to Present), Ovid EMBASE (1974 to present), and The Cochrane Library (Wiley). No language, publication date or article type restrictions were included in the search.

Data for the number of patients with cervical cancer with pathology confirmed invasion of uterovaginal prolapse was extracted from each study. Additionally, data on age, race, body mass index, family history criteria, tumour stage, tumour grade and histologic type were obtained where available. Data were entered into and exported from the Covidence online software program. Two independent investigators retrieved the data.

Study proportions were pooled and. Patient characteristics (i.e., age, tumour grade, histology, management and outcomes) were averaged over the studies to provide descriptive information. Publication bias was not assessed in this systematic review. All analyses were performed with the use of GraphPad Prism statistical software (Version 8.2.0)

## Results

## Discussion

Tumour invasion of prolapsed uterine corpus is uncommon among cervical cancer cases. Given the lack of reported cases and minimal literature on the topic, uterine corpus tumour invasion is not yet considered as a surgical-pathological risk factor, nor is it considered as a reliable marker for prognosis for women with cervical cancer [7]. A 2017 study by Matsuo *et al* [7] investigated 30 years of national data and identified 837 (4.9%) cases of uterine invasion among 17,074 cases of early-stage cervical cancer who underwent hysterectomy, however, none exhibited invasion of pelvic organ prolapse. This study demonstrated that invasion is typically associated with older age, non-squamous histology, high-grade tumours, larger tumour size and node status. Additionally, invasion was associated with a significant decrease in cause-specific survival when compared to cases without invasion [7]. Given decreased survival outcome among cervical cancer patients with tumour extension to adjacent organs, this study ultimately shows that uterine corpus invasion likely reflects aggressive tumour behaviour.

Cervical cancer invasion of complete uterovginal prolapse is exceedingly rare in developed countries. One of the first reported cases was in 1958 (Rocker *et al*), followed by reports in 1963 (Cabaniss *et al*) and 1972 (Daw *et al*) We performed a systematic review of PubMed, Embase, Medline and the Cochrane Library. A total of 13 reported cases of cancer association with uterovaginal prolapse were identified between 1990 and 2017. Reports ranged from different countries around the world including Austria, Brazil (two cases), France, Germany, India, Italy, Morocco, Portugal, Spain, Taiwan, Turkey and the United States. Table 1 lists each study with comparison of characteristics, management and outcomes [8–21].

The mean age at diagnosis among the reports is 74.4, ranging from 54 to 89 years of age. 13 of the 14 cases were squamous cell carcinoma of the cervix, one with verrucous features and one reported case of clear cell adenocarcinoma. Two cases presented with Stage I (14.2%), nine with Stage II (64.2%), three with Stage III (21.4%) and none with Stage IV. All had complete uterovaginal prolapse. Two cases did not undergo surgery (both stage IIIB), the third reported case of stage IIIB in Pardal *et al* [17] underwent total vaginal hysterectomy and partial colpectomy with colpoclesis. Eight cases underwent vaginal hysterectomy (57.1%), one underwent laparoscopic radical hysterectomy (7.1%). Three cases underwent lymphadenectomy (21.4%). Five surgeries addressed the pelvic organ prolapse (35.7%); one case performed perineorrhaphy, cystocele, enterocele and Gellhorn pessary placement, one case with intraabdominal uterosacral ligament suspension, and colpoclesis performed in three. Three cases did not receive chemotherapy or radiation. Ten cases received pelvic radiotherapy (71.4%), four received a combination of chemoradiation (28.5%)— all with cisplatin and one with 5-fluorouracil plus cisplatin. Patient outcomes were mixed. Seven patients were free of disease throughout follow-up (mean 21.5 months, ranging 6–60 months of follow-up reported). There were three deaths reported, two from disease (14.2%) at 3 and 12 months, and one from pulmonary embolism 20 days following surgery. Three cases did not report on the outcome and one case was last to follow up at 2 months.

Currently, there is no evidence-based management for cervical cancer associated with uterovaginal prolapse. Most reported cases in the literature suggest a radical vaginal hysterectomy with bilateral iliopelvic lymphadenectomy, external pelvic radiation and chemotherapy [17]. The decision for management must consider staging, the extent of spread, degree of procidentia and cystocele-rectocele involvement with risk of visceral injury following radiotherapy. The patient described above underwent PD-L1 testing with an attempt at alternative immunotherapy in hopes to optimise treatment. In June, 2018, the Food and Drug Administration approved pembrolizumab (Keytruda) with advanced, PD-L1-positive cervical cancer with progression while on or following chemotherapy. This decision was based on data from the Phase II KEYNOTE-158 trial which demonstrated an overall response rate of 17% (27% among patients with ≥27 weeks of follow-up) in advanced cervical squamous cell carcinomas [22]. This case further demonstrates the need for more literature surrounding cervical cancer with uterine invasion, especially associated with pelvic organ prolapse, in order to determine the most effective therapies.

## Conclusion

Overall, cervical cancer with invasion of uterovaginal prolapse is exceedingly rare in developed countries. There have been a total of 13 reported cases in the literature of cervical cancer association with uterovaginal prolapse between 1990 and 2017, each with various treatments and outcomes. There are no current management guidelines for cervical cancer with invasion of pelvic organ prolapse. As demonstrated from this study, management varies widely across the literature. However, a majority of cases underwent vaginal hysterectomy (57.1%) and pelvic radiotherapy (71.4%). The case reported above demonstrates the need to pursue additional biomarkers and therapies among this rare population. Especially, given the recent approval of pembrolizumab for advanced, PD-L1-positive cervical cancer with progression while on or following chemotherapy, PD-L1 testing and immunotherapy options should be considered. Further studies are necessary to further identify effective treatment strategies of uterovaginal prolapse associated with carcinoma of the cervix.

## Conflicts of interest

The authors have no conflicts of interest to disclose.

## Funding declaration

This study received no funding.

## Author contributions

Ryan M Kahn M.D., M.H.S: project development; data collection or management; data analysis; manuscript writing/editing.

Kiersten Craig M.D: project development; data collection or management; data analysis; manuscript writing/editing.

Tanaka J Dune M.D: project development; data collection or management; data analysis; manuscript writing/editing.

Kevin Holcomb: project development; data collection or management; data analysis; manuscript writing/editing.

Eloise Chapman-Davis M.D.: project development; data collection or management; data analysis; manuscript writing/editing.

Melissa K Frey M.D.: project development; data collection or management; data analysis; manuscript writing/editing.

## Figures and Tables

**Table 1.** Systematic review data of published cases of carcinoma of the cervix associated with procidentia.

Author	Age	Histology	Stage	Surgery	Chemotherapy/Radiotherapy	Outcome
Borgas de Silva 2001 [8]	69	Squamous cell carcinoma	IIA	Radical vaginal hysterectomy with bilateral salpingoo-ophorectomy and vaginal, parametrial resection	External pelvic radiotherapy at a total dose of 5,000 cGy fractionated over a period of 5 weeks	Free of disease at 2 years, lost to follow-up since
Borgas de Silva 2001	73	Squamous cell carcinoma	IIA	Radical vaginal hysterectomy	External pelvic radiotherapy at a total dose of 5,000 cGy fractionated over a period of 5 weeks	Alive with no signs of recurrence 2 years after surgery
Cabrera 2010 [9]	54	Clear cell adenocarcinoma	IB2	Laparoscopic radical hysterectomy with bilateral salpingo-oophorectomy, pelvic lymphadenectomy and para-aortic lymph node sampling	Adjuvant chemoradiotherapy, 46 Gy. Chemotherapy based on 5-fluorouracil plus cisplatin	Alive with no signs of relapse 10 months after treatment
Cheung 2012 [10]	77	Squamous cell carcinoma	IIB	Total excision	Post-operative tomoradiation of 6,000 cGy in 30 fractions to the pelvis	Not reported
Chung 2018 [11]	67	Squamous cell carcinoma	IIA2	Total vaginal hysterectomy, exploratory laparotomy, bilateral Salpingooophorectomy, cancer staging, and intraabdominal uterosacral ligament suspension	Adjuvant chemoradiation	Not reported
Dane 2009 [12]	89	Squamous cell carcinoma (Verrucous)	IIA	Resection of the vagina and parametria	Did not receive	Free of disease at 6 months of follow up
Dawkins 2018 [13]	72	Squamous cell carcinoma	IIA2	Perineorrhaphy, cystocele, enterocele repair, Gellhorn pessary placement	External beam radiation therapy, vaginal brachytherapy. Chemotherapy with cisplatin	Free of disease at 15 months
El-Abbassi 2017 [14]	79	Squamous cell carcinoma	IIIB	Did not undergo surgery	Palliative Chemotherapy	Dead of Disease at 3 months
Kriplani 1995 [15]	60	Squamous cell carcinoma	IIIB	Did not undergo surgery	Radiotherapy for a total of 50 Grays, 27 fractions over 5.5 weeks	Subsequent visit 2 months after completion of radiotherapy did not reveal any disease. Intracavitary radiotherapy was planned but the patient did not return for follow-up
Loizzi 2010 [16]	86	Squamous cell carcinoma	IIA	Vaginal hysterectomy with upper vaginectomy in spinal anesthesia due to poor performance status	Did not receive	Died of pulmonary embolism 20 days after surgery
Pardal 2015 [17]	74	Squamous cell carcinoma	IIIB	Vaginal hysterectomy plus open bilateral iliopelvic lymphadenectomy	Pelvic external beam radiotherapy 50Gy/25F plus vaginal brachytherapy 3×7Gy) and chemotherapy with cisplatin (40 mg/m^2^/weekly). Disease progressed, palliative therapy with paclitaxel (175 mg/m^2^) plus carboplatin (AUC5) every 3weeks.	Twelve months after the diagnosis the patient was admitted in the hospital due to an insidious onset of altered mental status and end-life care performed
Reimer 2008 [18]	73	Squamous cell carcinoma	IIA	Total vaginal hysterectomy and partial colpectomy with colpocleisis	Combined pelvic radiotherapy with cisplatin	After five years of follow up, no disease recurrent and pelvic floor stability
Reisenauer 2017 [19]	81	Squamous cell carcinoma	IIA	Vaginal radical hysterectomy, salpingo-oophorectomy, laparoscopic sentinel pelvic lymphadenectomy, Le Fort colpoclesis	Did not receive	Not reported
Vieillefosse 2014 [20]	87	Squamous cell carcinoma	IB2	Rouhier proceure: Modified LeFort colopcleisis with hysterectomy	External pelvic radiotherapy (dosing not reported)	Free of disease at 1 year
Cabanis 1963 [21]	68	Squamous cell carcinoma	IIB	---	---	Alive with disease at 8 months, lung metastasis
